# Early Recollections from 1846 to 1863

**Published:** 1909-11-13

**Authors:** B. Browning

**Affiliations:** Fellow of the Royal Sanitary Institute, etc.


### "'TIS SIXTY YEARS SINCE."

### EARLY RECOLLECTIONS FROM 1846 TO 1863.

By B. Browning, R.N., D.P.H.; Fellow of the Royal Sanitary Institute, etc.

This phrase may possibly be recognised as a prefix to the first of the Waverley novels, which, alas for the modern reader's taste, were formerly so much more popular than now. Without daring to compare my recollections with Sir Walter's, I venture to hope that mine will be deemed not altogether uninteresting. Craving your indulgence for necessary egotism, since Sir William Church has preceded me with an account of London hospitals as they were twelve years after my time, I will commence by reminding you of the discovery of chloroform in 1846—a time when almost every country surgeon and apothecary was experimenting with it, and, marvellous to say, with very few fatal results.

### Those Stirring Times.

That was a stirring era, both in matters political and medical. The Corn Laws were repealed, and anæsthesia was successfully produced. Really, one theme excited almost as much interest as the other; the *political* was at once firmly established; the *medical* very slightly so, especially in the provinces and colonies. Chloroform, in order to annul pain in all operations, as well as in dentistry and midwifery, was at first freely used by practically every practitioner, and talked of by the whole laity. Soon both parties took strong objections to it, and this repugnance continued for quite twenty years, and is perhaps not altogether extinct now. For example, the Principal Medical Officer of our troops in the Crimea (1854-6) formally forbade its being given, on the ground that "The smart of the knife was beneficial"! It was also omitted at the Royal Naval Hospital, Plymouth, even for capital operations, and most private practitioners declined to administer it in midwifery, although earnestly begged to do so by patients.

### Medical Education Then.

The professional education of the budding English medico was in many ways somewhat simpler than now; so were his studies and necessary expenses. As a rule, with the exception of the Apothecaries' Hall and Universities of Oxford, Cambridge, London, and Durham, no literary acquirements were necessary, beyond some knowledge of the "three R's"; but an apprenticeship of at least five years' duration, two of which must be passed under the tuition of a "master" (himself not necessarily a Diplomate), either in town or country, and three subsequently at a School of Medicine, was sternly demanded. The apprenticeship fee varied, but was usually from one hundred to three hundred guineas in all, according to the professional status of the "master," who, when accepting the pupil, and receiving him into his family, in most cases derived substantial help and diminution of professional expenses (in addition to the honorarium) by so doing. I have known a surgeon in a large provincial city who invariably, in his private practice, carried out every great operation (and he was frequently called on to perform them), solely with the help of his apprentices, and without any qualified aid. After the apprentice's first year not only the routine work of the "surgery," which involved a fairly practical acquirement of pharmacy, was usually entrusted to him, but he had often the almost entire charge of those parish patients who had to be visited at their homes. Part of his duties, in addition to compounding the medicines, doing various menial tasks, and sometimes helping the groom, was also to phlebotomise those bucolics who came regularly to "be *blooded*, every spring and fall," and occasionally he received a fee of five shillings for doing so; now and then, but very rarely, was he shown the difference between an aneurysmal varix and a varicose aneurysm! On commencing his "walking the hospital" therefore, he had some rudimentary knowledge of prescribing and minor surgical work, but knew hardly anything of Anatomy, Physiology, or Rational Medicine.

### Hospital Students and College Lectures.

In the "'forties to 'sixties" of Queen Victoria's reign, the prevalent recommendation of my Hospital Professors tended towards "practical" rather than "theoretical" study; "bedside work, not book-work" was their motto. At University College Hospital, the Professor of Clinical Surgery was wont to say, "Don't waste your time over a microscope, leave test tubes alone: *live* in the wards, if you want to know your profession." In the lecture rooms, the Professor of Physiology only gave *one* microscopic demonstration, that of the blood-circulation, as seen in the living frog's foot. The structure of tissues was illustrated by drawings, and sometimes by blackboard sketches; and the Professor of Pathology merely recommended his audience to learn the minute anatomy of the kidney by microscopic demonstration, which he did not give in the class-room, though often he would show students pus, various urinary deposits, and renal casts, under the microscope, when going round the wards. The clinical instruction given by the various professors was thorough, painstaking, and practical: I doubt if it has been surpassed in these respects by their successors.

### Hospital Work.

Our general hospital routine was much as that lately described by Sir William Church, but I am sure that more care was taken, both in the medical and surgical wards, as to the timely and instant segregation of fever, erysipelatous, or gangrenous patients, than at "Bart's.," and cases thus affected were at once removed elsewhere, if possible. The entire number of lectures delivered daily averaged six in all; but few men attended more than three courses in one term; and, in fact, we were discouraged from doing so. The hospital and professorial staff had a firm and warm hold on the respect and affection of the students, although some men, perhaps not unnaturally, objected to their discountenance of all athletic games and recreation, which, indeed, were absolutely forbidden in the spacious College grounds. As no Students' Athletic Union then existed in London, cricket, tennis, or boating was only practicable under very great difficulties. Football was not then in vogue, except amongst old Etonians and Rugbeians.

Our young fellows' ethical standard was as high as any I have ever known elsewhere, and any approach to the characters of Bob Sawyer or Ben Allen never, I am positive, existed amongst them. Though from the character of its entourage, street and factory accidents were comparatively rare at U.C.H., yet as it was really, as well as nominally, the "North London Hospital," surgical cases frequently demanded admission, and we revelled in fractures! The modern immediate fixed treatment of most of these last was anticipated and actually commenced there, by Sir John Erichsen, long before other hospital surgeons had attempted it, and even before some examiners of the Royal College of Surgeons had heard of it, as was proved in my examination by them.

The germs of Listerism were also brought to light by Erichsen and Richard Quain, when I was a dresser to them; for in their practice nothing but scrupulously clean hot and cold water-dressings were permitted, the fluid being mixed with some antiseptic when advisable, and such Abernethian remedies as ointments and poultices were relegated to the limbo of the past, though they still survived at "Bart's." Sir Wm. Jenner's researches on enteric and other fevers were also carried out during my student days, and the commencement of practical hygiene by the labours of Professor Edmond A. Parkes, later of Netley and cosmopolitan fame, dates from that time.

### Our Nurses.

I may now refer to the nurses, who in no wise resembled Mmes. "Sairey Gamp" and "Betsey Prig." There were but two nurses—termed "Sister" and "Nurse"—in each ward, the cleaning and other housework being done by wardmaids, and better trained nurses, by the then approved standard, than ours at U.C.H., could not be found, nor any more appreciated by their patients. Kind, middle-aged, self-denying, intelligent, experienced, zealous women they all were. The usual ward work except taking temperatures, a process then unknown, and attending to the personal needs of the patients, now performed by the hospital nurse of to-day, was carried out by the ward clerks and dressers, two being always on duty for each physician and surgeon, under the direction of the house-physicians and house-surgeons, and all supervised by the "resident hospital physician," a young qualified man of high professional attainments; his subordinates were not necessarily qualified, but simply appointed at the discretion of the chief under whom they were to serve. There was not then so much "hospital abuse" complained of by the hard-working G.P. as there is now; patients able to pay rarely defrauded him by means of a hospital ticket.

### Examinations.

At the termination of three years' London study the ordeal of the "College and Hall exam's" had to be undergone by the average student; these were short, sharp, and often decisive. The College examination, an oral one, was deemed the more severe; it lasted an hour, was divided into four *mauvais quarts d'heures*, engineered at four tables, each worked by two examiners, members of the College Council. Two tables were for anatomy, two for surgery. As the examination-room clock marked the quarters, it sounded its bell, when each examinee rose and passed on to the next table, and so on till the eight examiners had been visited, unless he heard the fatal formula pronounced: "You need not wait, sir," in which case he forthwith fled to the secretary's office, reclaimed his 20 guineas fee, and departed. A fresh martyr succeeded him, and those who had escaped his ill-fortune retired to the "funking-room," where they waited in varying spirits until the last man had been dealt with, and the fortunate ones were called in to receive the approval and congratulations of the president, whilst the unfortunates, "the goats," were simply granted liberty to depart by the secretary. As a rule, the happy "sheep" then adjourned to the "Black Jack" in Portugal Street and drank each other's health from the black jacks kept there for that purpose, after signing their names in a register which was said to date back for more than 100 years. The number examined was usually from 12 to 18, and the examination took place almost every Thursday during the winter and spring sessions.

### Passing the Hall.

The examination for the L.S.A. diploma was also oral, and held every week in the winter and spring sessions; it was confined to pharmacy, medicine, and midwifery, surgery being left severely alone. It lasted for an hour and a half, and was carried out on much the same lines as that in Lincoln's Inn Fields, with the exceptions that its fee was but six guineas, and that the examiners, not being hospital teachers, but general practitioners only, and also very rarely possessing more than the College or Hall Diplomas, were not so much dreaded. There was, however, a prevalent idea that it was very advisable to state the old-fashioned practice, and not to mention new-fangled "notions," if you wished to be sure of passing; consequently men going in for the Hall frequently cultivated the society of freshmen from the country, so as to recall the treatment they had first been taught, and subsequently enjoined to forget.

Candidates for the London degrees, the College of Physicians, and F.R.C.S. diplomas were comparatively few, and were examined in a different manner, and with greatly more stringent tests: much longer study, and larger fees were also exacted.

The cost of necessary lectures and hospital practice for admission to both College and Hall examinations was about ninety guineas in all; for the College alone, seventy-five guineas; for the Hall, sixty guineas.

### Memoirs of Practice—1850-1863.

After being graced with a diploma of some kind, though a non-diplomatist was sometimes found making a fair living, one usually entered into private practice by purchase or succession to an established connection; otherwise an appointment might be sought in the Naval, Army, or Colonial Medical Services. Appointments in the Public Services were very difficult to obtain; vacancies, except in war-time, were most infrequent, and interest, I imagine, had a great deal to do with success.

At the commencement of the Crimean war, the first time that Army Medical Commissions were obtained by competition, most of the best men in the London and Provincial Schools, many young graduates of the highest accomplishments, University scholars, gold medallists, and first-class prize-men, offered themselves to the Army Medical Director-General, and were refused on almost puerile grounds, generally as being physically unfit, when to most persons they would have appeared well-grown, and in rude health. The Navy was not then so much preferred; but there also the supply largely exceeded the demand. However, after the first Crimean winter both Departments immediately discovered their error, and did their utmost to obtain the enlistment of those whom they had previously rejected, but with little success, except by attracting some few men who followed their great leader Parkes to Scutari and the front of Sebastopol, and thus aided their military and naval *confrères* to save some valuable lives, after a frightful number had been stupidly and uselessly wasted.

### The Crimean Days.

To realise the advances that, owing to Lord Herbert of Lea, Miss Nightingale, Parkes, and Lister, surgery has made since those far-off days I remember, one must have witnessed, as I did, the debarkation of sick and wounded from the Crimea and their state on reception into hospital. Sufferers from various fevers, diarrhœa, dysentery, amputation and other wounds, fractures, etc., had all been crowded together in a badly ventilated, ill-lighted, chilly merchant ship's lower deck, and though given the best attention which a harassed and feebly-staffed medical officer in charge could arrange, had undergone hardships which now would never be permitted, and which were even then quite preventable. The condition of these poor fellows was often almost too horrible for description, and a second amputation, higher up the limb than the first, was sometimes necessitated subsequently, from the effects of the inferno they had laid and suffered in during their fortnight's passage home.

Having been honoured with a commission in the Royal Navy as assistant-surgeon during the latter part of the Crimean war, I thought that the Home Naval hospitals were then greatly understaffed, and their chiefs not quite up to the teaching of Jenner and Erichsen, but that their devotion and rigid attention to their arduous duties could not be surpassed; and I consider a Naval hospital of to-day an up-to-date model; even in the "'fifties" it was so in many, if not all, respects. Proceeding in the last of the old sailing frigates to Australia, and afterwards to New Zealand, I found three Universities and hospitals (the latter not very good ones), but no medical schools or clinical teaching, all (and there were not many) Antipodean students being accordingly obliged to study in Europe or America.

### At the Antipodes.

As most surgeons settled in the Southern Hemisphere had quitted the Mother-Country very many years ago, their practice was generally a replica of that of my grandfather's period—bleeding, cupping, blistering, and tartar emetic being their sheet-anchors; their surgery, though rough sometimes, was effectual. Whilst in the South Sea Islands I observed that some of the natives' remedies seemed equal, if not superior, to those of the missionaries. Here, also, I had many opportunities of practising the operative surgery I had been taught at home, and with no fear of a Coroner's inquest before my eyes. Latterly, whilst engaged in the New Zealand war of 1860, it was instructive to notice how our men, wounded never by artillery fire, but only by bullets and tomahawks, rapidly recovered, the wounds usually healing by first intention, water-dressing and the purest air being the chief factors in their well-doing; but this was even more strongly marked in the case of the few wounded Maori whom I saw.

Being transferred from the South Pacific to the Mediterranean, nearly all parts of which classic sea I visited, I was interested by the firmly rooted and seemingly well-grounded belief of the population of each country in the communicability of pulmonary consumption—a creed which, from what I witnessed in various ports, and on board H.M.S. *St. Jean d'Acre*, I adopted, after much reflection Although when I brought my new views on this theme before a well-known London medical society—the first man, I believe, to promulgate it—I was hardly accorded a hearing, and almost laughed to scorn, I have lived since then to see the doctrine universally accepted, and to witness the modern recognition of the value of preventive medicine, which, as one of its early and humble pioneers, I am proud to find now taught in most schools of medicine, after having been long ignored by all.

